# CityJSON in QGIS: Development of an open‐source plugin

**DOI:** 10.1111/tgis.12657

**Published:** 2020-06-24

**Authors:** Stelios Vitalis, Ken Arroyo Ohori, Jantien Stoter

**Affiliations:** ^1^ 3D Geoinformation Group Delft University of Technology Delft the Netherlands

## Abstract

When QGIS 3.0 was released in 2018, it added support for 3D visualisation. At the same time, CityJSON has been developing as an easy‐to‐use JavaScript Object Notation (JSON) encoding for 3D city models using the CityGML 2.0 data model. Together, this opened the possibility to support semantic 3D city models in the popular open‐source GIS software for the first time. In order to add support for 3D city models in QGIS, we have developed a plugin that enables CityJSON datasets to be loaded. The plugin parses a CityJSON file and analyses its tree structure to identify all city objects. Then, the geometry and attributes of every city object are transformed into QGIS features and divided into layers according to user preferences. CityJSON parsing was proven to be straightforward and consistent when tested against several open datasets. One of the biggest challenges we faced, though, was mapping CityJSON’s hierarchical data structure to the relational model of QGIS. We undertook this issue by providing various methods on how geometries from the model are loaded as QGIS features. We intend to use the plugin for educational purposes in our university and we believe it can be proven a worthy tool for researchers and practitioners.

## 1 INTRODUCTION

For many years three‐dimensional (3D) city models were mainly used for visualization purposes, but recently an increased demand for processing applications based on 3D urban data has been identified (Biljecki, Stoter, Ledoux, Zlatanova, & Çöltekin, 2015). For instance, such models have been used for applications such as 3D cadastres, facilities management and emergency response. As a result, more organizations, such as national agencies, are investing in and producing 3D city models, mainly offering them as open data (Ho, Crompvoets, & Stoter, 2018).

CityGML is the most popular standard for the representation of 3D city models (Open Geospatial Consortium, 2012). Although it has been available for more than a decade, there exists only a limited amount of support for handling CityGML files in GIS software. Furthermore, the support is mainly through proprietary tools, such as FME (https://www.safe.com/fme/fme‐desktop/) and ArcGIS (https://www.esri.com/arcgis/about‐arcgis), where the high cost of acquisition restricts access for researchers and practitioners. Meanwhile, loading CityGML successfully in open‐source GIS software requires complicated and fragile processes using external tools (https://3d.bk.tudelft.nl/svitalis/citygml/gdal/2017/07/24/messing‐around‐with‐citygml‐on‐gdal‐2.2.html).

One of the main reasons for CityGML’s poor interoperability is the complex and verbose nature of its main encoding, which is based on GML. For instance, there are at least 26 different ways of representing a simple square through GML (https://erouault.blogspot.com/2014/04/gml‐madness.html). Implementing software that can robustly parse such a data format is extremely challenging.

CityJSON has recently been introduced as a JSON encoding of CityGML’s data model in order to overcome such issues (Ledoux et al., 2019). The aim of the CityJSON data format is to provide support for almost all the features of CityGML, while at the same time maintaining a simple file structure that allows developers to easily manipulate CityJSON files.

Parallel to this, QGIS (QGIS Development Team, 2020) has added support for better manipulation and visualization of 3D data, available since version 3.0 (https://qgis.org/en/site/forusers/visualchangelog30/index.html). This opens up the possibility of support for 3D city models in a cross‐platform open‐source GIS with a wide user base in geoinformation education.

QGIS uses a relational data model where data are represented in a tabular form, which can easily represent information from many GIS data formats, such as Esri shapefiles and GeoJSON. Nevertheless, the mapping of a more complex data model, such as CityGML, to a relational data model is a non‐trivial process. Previous attempts to do so, such as 3dcitydb (Yao et al., 2018) which is a relational model to store CityGML data, result in a complicated database schema composed of multiple tables with complicated relationships. While such a solution provides a complete representation of the original data, it is practically impossible to manage in QGIS. That is due to the fact the GIS has very limited support for representing links between tables, which is an essential mechanism for representing object‐oriented concepts such as association and aggregation.

In order to support the loading of 3D city models in QGIS we have developed *CityJSON Loader* (https://plugins.qgis.org/plugins/CityJSON‐loader/), a plugin that enables CityJSON files to be loaded in the application. The software parses a CityJSON file and analyzes its tree structure to identify all city objects. Then the geometry and attributes of every city object are transformed into QGIS features. Finally, those features are stored in layers, which are then added to the current project with 3D rendering styles.^1^

We developed CityJSON Loader with the intention of making CityJSON (and, thus, 3D city models) more accessible to researchers and practitioners through general‐purpose GIS software (in this case, QGIS). Loading CityJSON in QGIS enables its extensive and powerful processing toolkit to be used on 3D city model data. Furthermore, we believe that QGIS can act as a transformation tool for 3D city models, due to its wide support for GIS formats through its utilization of GDAL.

In this article we provide documentation about the design choices, architecture, and *modus operandi* of CityJSON Loader. Mostly, we focus on our approach to solving the problem of mapping the CityGML data model, used by CityJSON, to the relational model of QGIS while maintaining its usability.

Section 2 provides an introduction to QGIS, a description of the CityJSON data format and related work regarding mapping CityGML data to a relational model. Section 3 describes the main architecture of the software developed, as well as implementation details. In addition, it explains how CityJSON data are mapped to the QGIS tabular structure. Section 4 presents the elements of the software that interact with the user. Section 5 demonstrates the use of CityJSON Loader with three open data sets, validating the software’s functionality against most use‐case scenarios. Finally, Section 7 presents our conclusions from developing and using the software, as well as suggestions for future improvement.

## 2 BACKGROUND

### 2.1 QGIS

QGIS (http://qgis.osgeo.org/) is open‐source GIS software which began as a project in 2002. It is governed by OSGEO (https://www.osgeo.org/) and is licensed under the GNU General Public License. While its initial goal was simply to develop a spatial data viewer, it has now evolved into a complete platform for the loading, transformation, and processing of spatial data (https://docs.qgis.org/3.10/en/docs/user_manual/).

Its main component is QGIS Desktop, a version of the software which runs on multiple desktop operating systems, including Linux, macOS, and Windows. The desktop application provides support for loading and saving to multiple vector and raster file formats through the Geospatial Data Abstraction Library (GDAL; GDAL/OGR Contributors, 2019), as well as additional providers for web services (e.g., Web Mapping Service and Web Feature Service).

Vector data are loaded into QGIS in a relational data model. Every feature has multiple attributes and a single geometry. Features are grouped in layers, so that all features of a layer have the same number of fields (similar to columns in a table of a relational database) and the same geometry type.

A plethora of processing algorithms are implemented in QGIS for overlaying, translating, and analyzing geospatial data. QGIS also provides support for the execution of geospatial processing algorithms from other tools, such as GDAL (Ose, 2018), GRASS (Lacaze, Dudek, & Picard, 2018), and SAGA (Passy & Théry, 2018). Furthermore, processing tools are being developed for multiple research purposes, such as exploratory spatial analysis (Gil, Varoudis, Karimi, & Penn, 2015), evaluation of aquatic ecosystems (Nielsen, Bolding, Hu, & Trolle, 2017), and classification of aerial and satellite imagery (Nielsen et al., 2017).

#### 2.1.1 QGIS technology and extensibility

QGIS is written in the C++ and Python programming languages. It is possible to extend its functionality by developing an application in either of those languages by utilizing its application programming interface (API).^2^

The QGIS Development Team maintains a central repository in order to distribute the application’s available plugins (https://plugins.qgis.org/plugins/). Developers can openly upload a new plugin, thus making it easily accessible to all QGIS users directly through the application.

### 2.2 CityJSON

CityJSON is a JSON encoding of the CityGML 2.0 data model (Ledoux et al., 2019). Its aim is to provide a more storage‐efficient and developer‐friendly way of storing 3D city models, while providing full compatibility with the CityGML standard.

**Figure 1:**
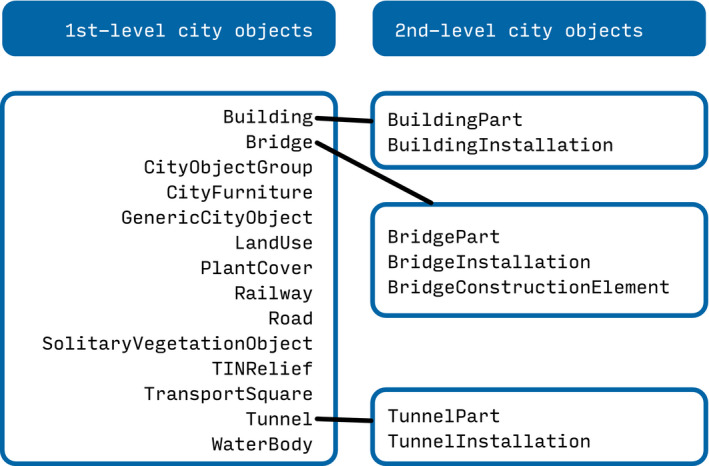
The available city object types according to the CityJSON specifications. A feature can be a first‐level object which describes an individual object, such as a building, or a second‐level object which describes part of a first‐level object (Source: Ledoux et al., 2019).

The data model provides definitions to describe urban objects (e.g., buildings and transportation infrastructure) and landscape features (e.g., terrain, vegetation, and water bodies). In CityJSON, every such feature is a JSON object with the respective type (Figure 1), a number of attributes, and a set of geometries. The multiple geometries of a city object correspond to different 3D representations of the object’s shape according to different levels of detail (LoDs).

A geometry object can be an object of any of the following types: MultiPoint, MultiLineString, MultiSurface, CompositeSurface, Solid, MultiSolid, or CompositeSolid. Intuitively, all geometric types (with the exception of MultiPoint and MultiLineString) can be considered as a set of polygons which describe the 3D shape of an object. What differs between the types of geometry is the shape’s semantic meaning; a Solid geometry is considered to be the volume bounded by the aforementioned polygons, while a MultiSurface or a CompositeSurface describes a surface object without a volumetric interior composed of those polygons. Every such polygon, which in CityJSON terminology is considered a surface, may also have an additional semantic meaning. This means that individual surfaces can have a semantic type (e.g., WallSurface, RoofSurface, and FloorSurface) with attributes that represent further information exclusive to that surface.

In addition to the previously mentioned explicit geometric types, CityJSON also provides support for the efficient storage of repeating geometries through the usage of geometry templates. This is done by defining a basic geometry as a template in the file and then referencing it as a GeometryInstance in a city object. For example, if a specific tree geometry is to be used multiple times in a city model, the tree geometry can be defined as a geometry template. Then, every tree city object can reference the template as a GeometryInstance, locating and transforming the original geometry in 3D space.

In order to achieve storage efficiency, CityJSON also supports a compression mechanism. When a CityJSON file is compressed, all coordinates are stored as integers and a basic transformation is defined for all of them. Therefore, in compressed files, all coordinates have to be scaled and translated (i.e., moved) based on the transformation defined. Through compression, CityJSON ensures that coordinates are normalized to a certain precision point.

CityGML currently has very limited support for metadata and most categories are only supported via the CityGML 3D Metadata ADE (https://github.com/tudelft3d/3D_Metadata_ADE), an application domain extension (Labetski, Kumar, Ledoux, & Stoter, 2018). In contrast to this, CityJSON has full support for metadata, both in conformance with ISO 19115, the metadata standard specifically for geographic information (ISO, 2014), and with further categories specific to 3D city models (e.g., LoD). Metadata can be at the city model level (e.g., presence of materials) or at the thematic module level (e.g., number of buildings per LoD).

#### 2.2.1 Software support

The main tool for managing CityJSON files is cjio (https://github.com/tudelft3d/cjio). This is a command‐line tool that allows users to query a file and access information about the model, including statistics and metadata. It is also capable of conducting multiple managing operations, including schema validation, compression/decompression, and the extraction of a subset of a CityJSON file. In addition to the command‐line interface, cjio provides a Python library for developers to manipulate CityJSON files and apply these operations on a data set.

citygmltools (https://github.com/citygml4j/citygml‐tools) is software developed in Java using the citygml4j (https://github.com/citygml4j/citygml4j) library. Although mainly developed as a set of tools to manipulate CityGML files, it provides support for parsing and saving CityJSON files as well. Therefore, it can be used to convert CityGML files to CityJSON and vice versa.

There are a variety of other open‐source tools that can manipulate and visualize CityJSON files. azul (https://github.com/tudelft3d/azul) is a viewer for 3D city models for macOS, which supports the loading of CityJSON. val3dity (https://github.com/tudelft3d/val3dity) can conduct the validation of 3D primitives in a CityJSON file. 3dfier (https://github.com/tudelft3d/3dfier) is software for the creation of 3D city models from 2D data sets, and it can produce CityJSON models specifically.

### 2.3 Object‐relational impedance mismatch

Mapping objects to tables, such as in the case of relational databases, is a common issue in computer science and practice (Keller, 1997). In the literature, this is further discussed as the general and inevitable issue of exchanging data between systems, which is referred to as “impedance mismatch” (Ireland, Bowers, Newton, & Waugh, 2009). This problem is formulated by Ireland et al. (2009) as an issue caused by differences between paradigms, languages, schemas and instances of object‐oriented representation of the universe of discourse, compared to a relational representation. In practice, this problem is normally addressed through the use of object‐relational mapping systems. Developers use such mechanisms in object‐oriented applications when they must use a relational database for persistence.

#### 2.3.1 Object‐relational mapping of CityGML

The CityGML data model is designed with an object‐oriented approach and formulated in UML. This causes a problem of impedance mismatch when trying to map CityGML data to a relational database. Normally, people address such issues by transforming the data from object‐oriented data models to relational data according to the needs of their specific use case using an extract, transform and load tool, such as FME (https://www.safe.com/fme/). Nevertheless, this requires a specific investigation of the data and is a process that has to be executed based on the particularities of every individual data set.

The problem of mapping CityGML to a relational model without loss of information has been addressed by 3dcitydb (Yao et al., 2018). 3dcitydb is a relational schema definition for representing 3D city model data as described by the CityGML data model. It also provides the tools for translating CityGML data to a database with the respective schema and vice versa.

Due to the fact that 3dcitydb aims to provide a complete representation of the original data model, the resulting schema is a complex set of tables and relationships. It is possible to use this schema as intermediate storage to load data in GIS software, such as QGIS, but in practice only a very small portion of the schema is necessary to visualize the required level of information. In addition, some essential functionality of GIS software, such as the identification of the attributes of a polygon, is non‐trivial as the information related to geometries is scattered in multiple tables. In most cases, though, such information could be easily simplified in such a way that the information and geometry are stored in one table, due to the fact that most data sets only use a very small portion of what the data model prescribes.

For example, it is easy to assume that intuitively a building could be represented by a set of attributes that represent its semantic information and the geometry of the building itself. Such a simple concept, though, is normally represented in 3dcitydb through multiple tables that represent the building, the geometry and any generic attributes through multiple rows in different tables.

## 3 SOFTWARE ARCHITECTURE

CityJSON Loader works as a plugin in QGIS which parses the content of a CityJSON file and maps it to QGIS features. As described in Section 2.2, we can assume that every city object is composed of multiple surfaces, which in QGIS would be perceived as 3D polygons. Therefore, the software’s main task is to create QGIS features with multi‐polygon geometries that represent the objects of the city model.

Mapping the CityJSON data model to the single tabular structure of QGIS layers, where every feature has a fixed number of fields and one geometry, is non‐trivial. A simple approach is to assume that every city object can be mapped to a feature in a QGIS layer. While this might be a good representation for simple city objects with a single geometry (i.e., one LoD), it is not sufficient for all cases. This is due to the fact that a city object can have more than one geometry, due to multiple LoDs. Merging multiple LoD geometries into one multi‐polygon feature would render the visualization, or any geometric processing, useless. Furthermore, CityJSON’s semantic surfaces mechanism offers the possibility of attaching individual information to every surface. If all surfaces of a geometry were merged into one multi‐polygon QGIS feature, then all semantic information regarding the surfaces would be lost.

### 3.1 Mapping of surfaces to polygons

**Figure 2:**
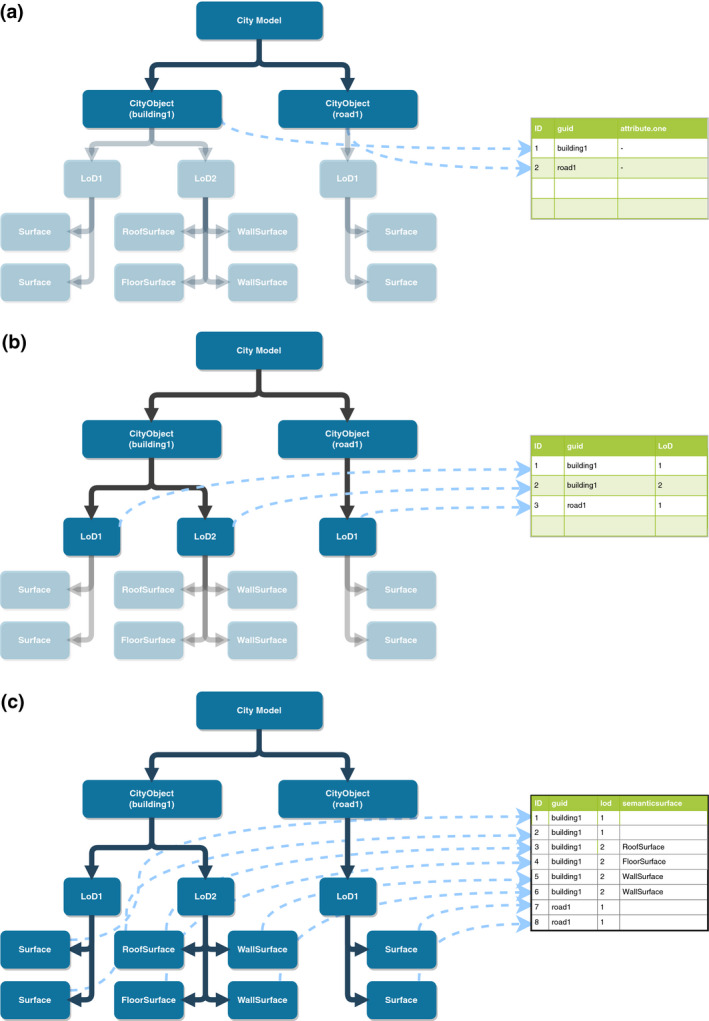
The different mappings between geometries of the city model and the resulting QGIS features. (a) Simple mapping will create a QGIS feature for every city object in the model. (b) If the user chooses to load LoDs (but not semantic surfaces), then every city object will have to be represented with multiple features in QGIS. In this case, the semantic information (i.e., attributes) will be repeated. (c) If the user chooses to load semantic surfaces, then every surface of a city object will be represented as an individual feature in QGIS. Similar to the LoD case, the semantic information (i.e., attributes) of the city object will be repeated for every surface

In order to tackle these problems, CityJSON Loader provides various options for different mappings between city objects and QGIS features (Figure 2). This means that, depending on the user’s choice, a QGIS feature can correspond to: a city object, where all geometries are represented by one multi‐polygon (Figure 2a); a city object’s LoD, where a city object is divided into multiple features (one feature per LoD geometry; (Figure 2b)); or a semantic surface of a city object, where every surface of a city object forms its own QGIS polygon feature (Figure 2c).

### 3.2 Division of features into layers

The software is also designed in such a way that the resulting features can be split into different layers according to: their object type (e.g., Building, Road, and Terrain), or their LoD (as soon as the LoD geometries are loaded as individual features). Those options can be combined, so that, for instance, a layer is created for every LoD of every object type. This allows users to visualize and process different LoDs separately in multi‐LoD data sets. In addition, by dividing features by object type, it is easier to style and manage objects of different kinds.^3^

### 3.3 General loading process

**Figure 3:**
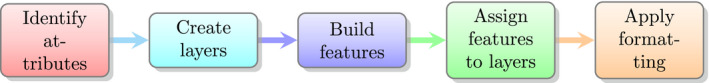
The main workflow of the CityJSON loading process by the software

The software conducts the loading process in five individual steps (Figure 3). The workflow is structured in such a way so that the different mappings or division of features to layer can be applied in a flexible way. This can be achieved by changing the behavior of every step based on the user’s requirements.

First, the software iterates through the model’s city objects to identify all attributes contained in the data set. During this step, a list of the attributes is constructed for use in creating the QGIS layers.

In the second step, the QGIS data sources and layers are created. This is done by enumerating the attributes identified in the first step. Additionally, at this step certain supplementary fields might be created: one for the LoD of the feature, in cases where the user chooses to load LoD geometries as individual features; and one for the semantic surfaces, in cases where the user wishes to load the geometry of the surfaces of an object.

During the third step, the actual QGIS features are built. This is done following the mappings described in Section 3.1, which means that features are built by city object, by LoD, or by semantic surface.

At the fourth step, the features created are assigned to the layers created during the second step. This is done based on the features’ attributes and the desired division. For instance, if the user chooses to have different LoD features in individual layers, then the respective LoD field is used by the software to decide which layer the feature is assigned to.

Finally, the layers are added to the active QGIS project. Some basic formatting is applied to the layers during this step. The software enables 3D rendering for every layer that was created by applying the same color style as the 2D view.^4^ Depending on user preferences, a rule‐based styling can be applied in 3D so that features are colored based on their semantic surface type (e.g., red for RoofSurface and white for WallSurface).

### 3.4 Implementation

CityJSON Loader is written in Python 3. It utilizes the QGIS API in order to communicate with the host application. It was originally developed in QGIS 3.2 and is compatible with all version of QGIS since 3.0.

**Figure 4:**
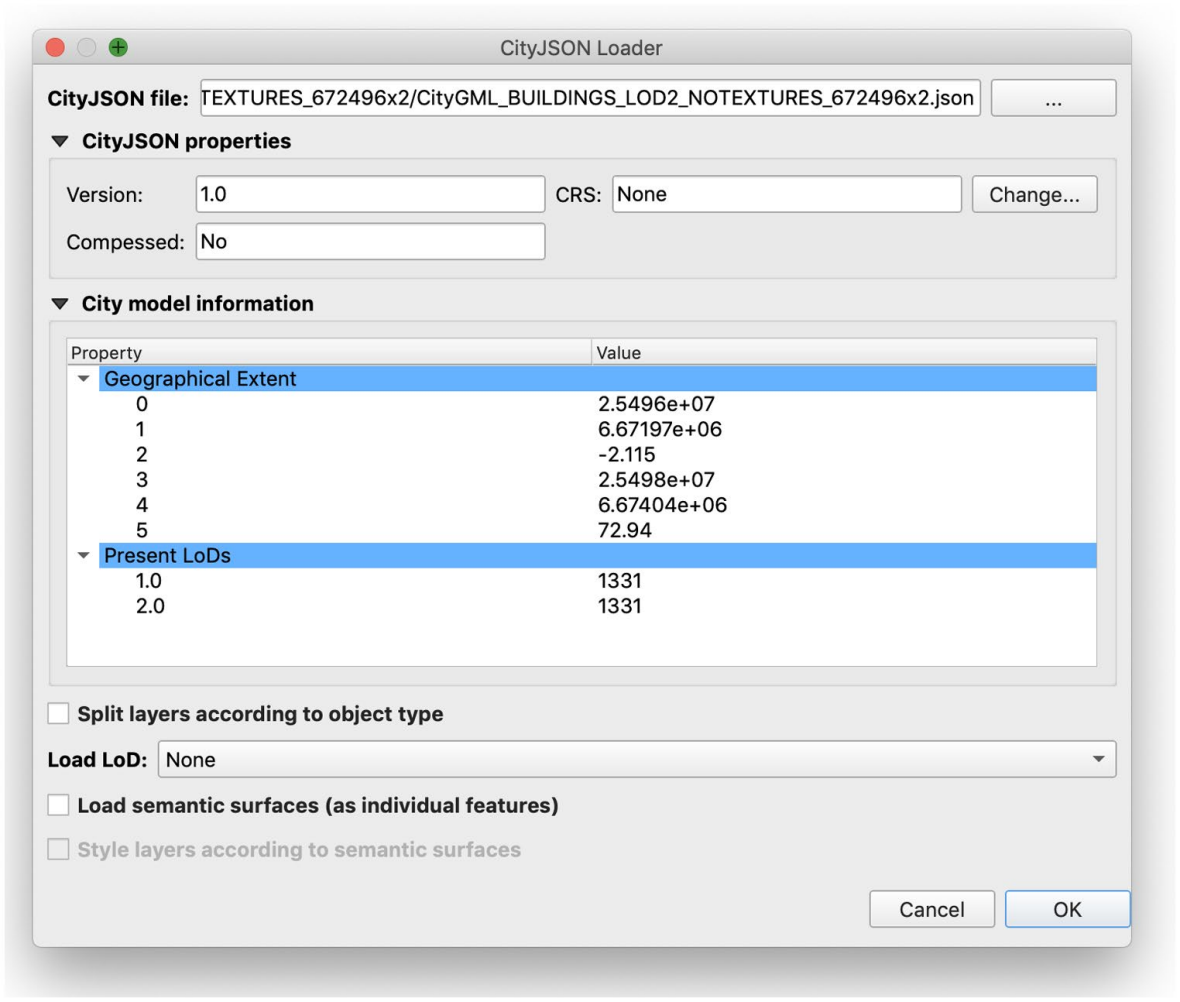
The main dialog providing options for loading a CityJSON file

The software’s interface uses Qt for all user interface elements. The loading dialog (Figure 4) is designed in Qt Creator (https://doc.qt.io/qtcreator/). JSON parsing and manipulation of a city model’s data is done through the json library provided by Python. There are no additional software requirements for the plugin to work, as all aforementioned libraries are provided by the QGIS installation.

The analysis and processing of the semantic and geometric information are done by the core library of CityJSON Loader. The core library was designed in order to implement the process described in Section 3.3. Certain software design patterns are followed in order to allow the software’s core library to be extensible. The main workflow is implemented depending on the strategy pattern, thus making the individual steps variable. In addition, the functionality of every step can be adjusted by using the decorator pattern. For instance, a basic class creates the layers by adding fields for the attributes identified in the city model. This behavior can be enhanced by applying decorative classes that will also create an lod or semanticsurface field if necessary, based on the user’s input.

## 4 USER INTERACTION

The interaction between the software and the user mainly takes place through the loading dialog (Figure 4). This dialog can be initiated by the shortcut provided or the appropriate menu entry found in the QGIS Vector menu.

In the dialog, the user can select a file using the operating system’s file explorer by pressing the top‐right button. After a CityJSON file is selected, the main information regarding the file is shown in the dialog.

The *CityJSON properties* group shows the CityJSON version of the file, the coordinate reference system (CRS) of the city model and whether the CityJSON file is compressed or not. The CRS field is filled according to the city model’s metadata, if a coordinate system is defined. Nevertheless, the user can set another CRS if none is found or if they prefer to load the layers with a different one.

The *City model information* group presents the metadata information contained in the file. The information is shown in key–value pairs, while nested information is also shown and can be collapsed or expanded.

Finally, the dialog provides a number of user options which impact the loading process.

- The *Split layer according to object type* option splits features of different object types (e.g., Building and Terrain) into different layers.
- The *Load LoD* option provides two choices: *As Attribute*, which loads different LoD geometries as individual features and adds a lod field; or *As Layers*, which splits those features into individual layers.
- The *Load semantic surfaces (as individual features)* further divides city objects by semantic surface and adds a semanticsurface field.
- The *Style layers according to semantic surfaces* adds a rule‐based style to all new layers so that the features are colored according to their semantic type (e.g., every WallSurface would be white and every RoofSurface would be red).^5^

## 5 EXAMPLES

In this section we provide examples of using CityJSON Loader with three open data sets:

- Den Haag, which contains LoD2 buildings and terrain;
- Helsinki, which contains LoD1 and LoD2 buildings; and
- a data set representing the landscape around a railway (from a data set that was originally introduced to demonstrate a plethora of CityGML 2.0 city object types).

### 5.1 Den Haag

The Den Haag data set is provided by the municipality of The Hague through its web data platform (https://denhaag.dataplatform.nl/#/data/756d6fe7‐f334‐4ced‐af13‐61d612d92b1a) and contains buildings and the terrain for the city. This model was produced in 2010 based on the national registration of buildings (BAG (https://zakelijk.kadaster.nl/bag)) and areal images acquired that year. The complete data set is split across 152 tiles which contain overall around 112,500 buildings in The Hague and neighboring municipalities.

The file tested with CityJSON Loader is a tile which contains 2,498 city objects, of which one is a TINRelief and the remainder are Building and BuildingPart objects. It was converted from CityGML to CityJSON using citygmtools. It contains 1,991 LoD2 geometries of MultiSurface and CompositeSurface types, with the presence of the semantic surfaces RoofSurface, WallSurface, and GroundSurface. We used this file in order to evaluate the functionality of the software against what is considered a “typical” 3D city model, mostly composed of buildings and terrain with LoD2 geometries and semantic surfaces.

**Figure 5:**
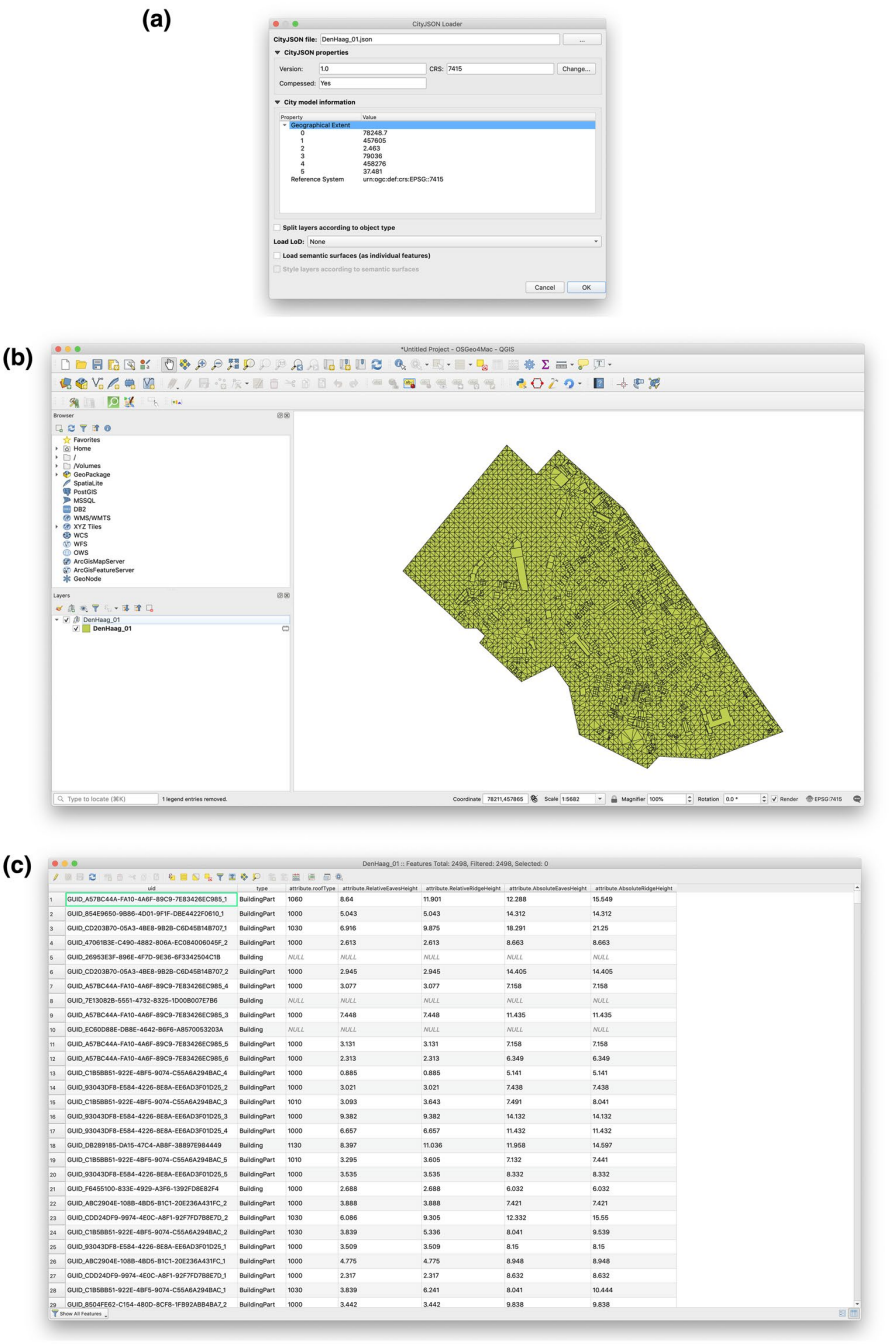
Screenshots of the application while initially loading the Den Haag data set with a simple mapping. (a) In the dialog we selected the CityJSON file containing the Den Haag data set. The CRS was successfully parsed from the relevant metadata property (*Reference System*). (b) Given the options we provided, only one layer was loaded in the active project containing all city objects, regardless of their object type. (c) The attributes table window shows that all semantic information (attributes) for the 2,498 city objects are loaded successfully

**Figure 6:**
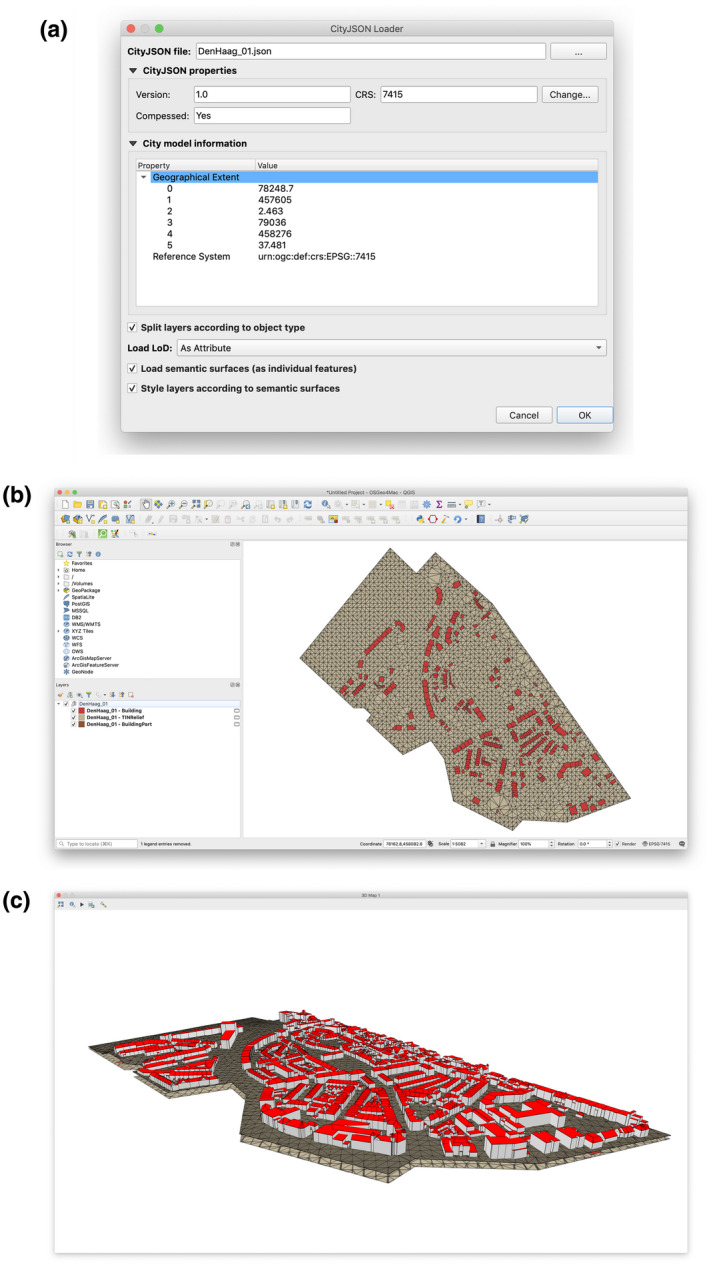
Screenshots of the application while loading the Den Haag data set with mapping features by semantic surface. (a) In the dialog we selected the CityJSON file containing the Den Haag data set. Splitting objects by type, loading LoDs as attributes and mapping features per semantic surfaces is selected. In addition, semantic surface formatting rules are enabled. (b) Given the options we provided, three layers were created: one per object type (Building, BuildingPart and TINRelief). (c) The 3D view visualizes the surfaces with rule‐based coloring: white for WallSurface and red for RoofSurface

We loaded the data set with two different set of options: one with simple mapping (one feature per city object) and all features loaded in one layer (Figure 5); and one with semantic surfaces mapping (one feature per semantic surface) and features divided by object type (Figure 6). The tests show the ability of CityJSON Loader to parse a simple 3D city model. Through the simple 2D and 3D view, and by using the attributes table, we can inspect the 3D city model’s semantic information. Both 2D and 3D identification also work.

### 5.2 Helsinki

The Helsinki city information model has been developed by the municipality of Helsinki in order to enable users to perform analyses such as calculating energy consumption and greenhouse gases (https://hri.fi/data/en_GB/dataset/helsingin‐3d‐kaupunkimalli). It is available as open data and can be downloaded through the dedicated web portal (https://kartta.hel.fi/3d/) or as CityGML tiles (http://3d.hel.ninja/data/citygml/). The city model contains buildings of the area with LoD1 and LoD2 geometries. The model uses the ETRS‐GK25 plane coordinate system and the N2000 height system (namely, EPSG:3132).

**Figure 7:**
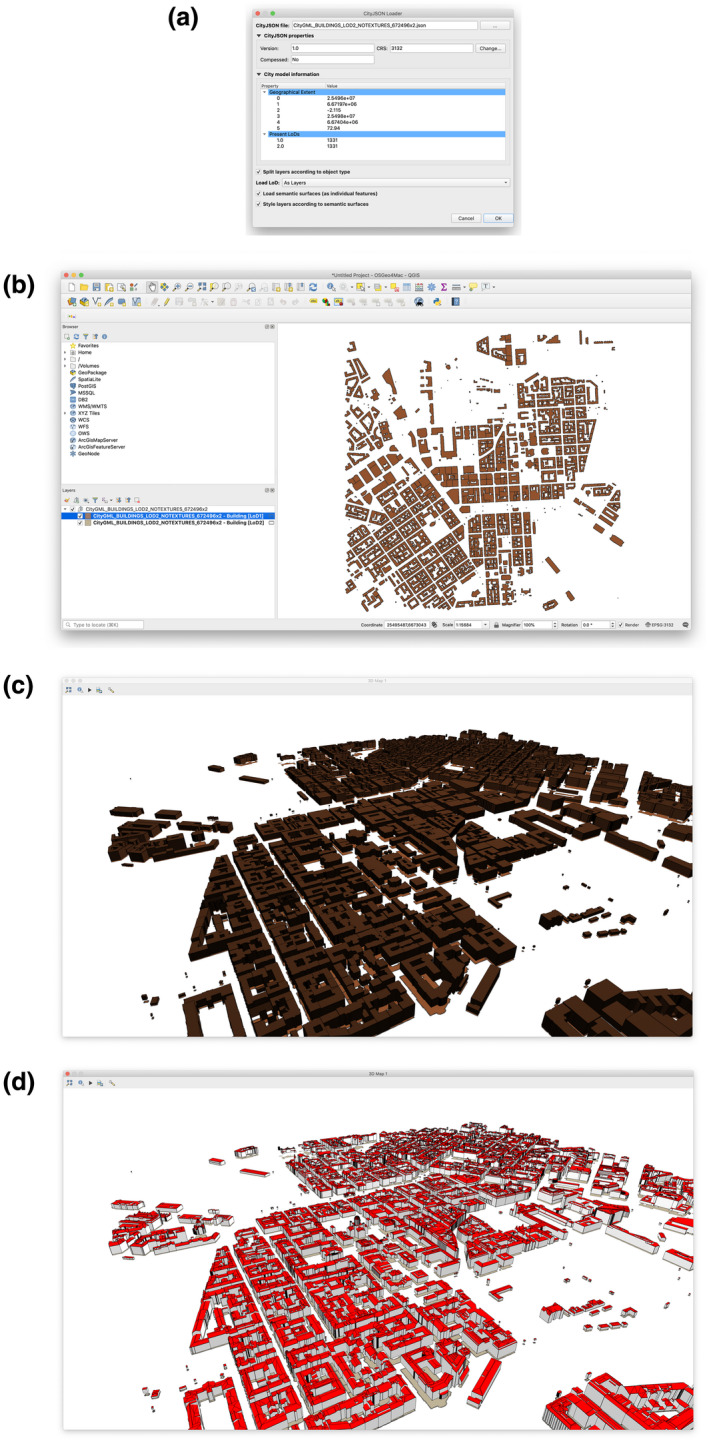
Screenshots of the application while loading a tile of the Helsinki data set. (a) In the dialog we selected the CityJSON file containing the Helsinki data set. According to the metadata, both LoD1 and LoD2 are present (for 1,331 objects). There is no CRS defined in the metadata, but we chose ETRS‐GK25/N2000 (EPSG:3132) as mentioned on the data set website. (b) Given the options we provided, two layers were created, one for the Building objects of each LoD. (c) The 3D view of LoD1 shows the prismatic geometries of the 1,331 buildings in the data set. (d) The 3D view of LoD2 shows the surfaces with rule‐based coloring: white for WallSurface and red for RoofSurface.

For our tests, we downloaded a CityGML tile of 1,331 Building objects. The CityGML file was converted to CityJSON using citygmltools and then tested with CityJSON Loader (Figure 7).

This CityJSON file was selected in order to validate the functionality of the software when working with multi‐LoD data sets. In this case, both LoD1 and LoD2 geometries are present, therefore the mechanism of splitting city objects by LoD (preferably to layers) was necessary in order to properly visualize the data.

#### 5.2.1 Railway demo

The railway demo data set is a 3D city model that was procedurally produced in order to demonstrate most CityGML 2.0 city object types. It contains 121 city objects of 14 different object types. It is composed of 105 MultiSurface and CompositeSurface geometries at LoD3 with semantic surfaces and utilizes the mechanism of GeometryInstance.

**Figure 8:**
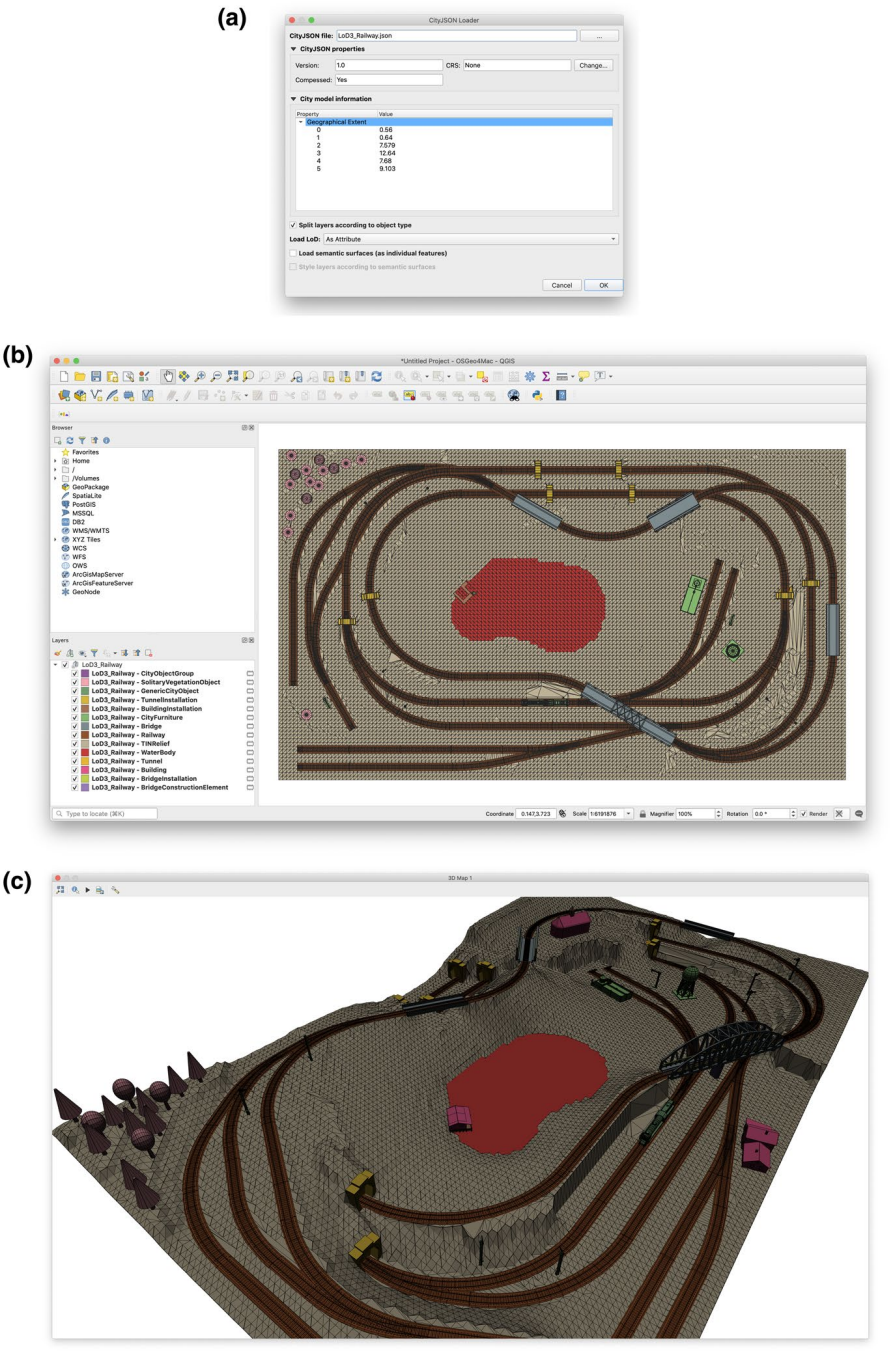
Screenshots of the application while loading the railway data set. (a) In the dialog we selected the CityJSON file containing the railway data set. As there is no CRS defined in the metadata and the geographic extents seem to be arbitrary, we defined no CRS. Splitting objects by type and loading LoDs as attributes is selected. (b) Given the options we provided, the city objects of all 14 types were loaded. Since there is no geometry, the CityObjectGroup layer is empty, as expected. Nevertheless, all other features were loaded successfully, including the GeometryInstance objects (mainly trees which are of SolitaryVegetationObject type). (c) The 3D view visualizes the objects with similar colors to the 2D view (as there are no semantic surfaces loaded). We also validated that the trees that are represented as GeometryInstance geometries are successfully loaded and rendered

We tested this CityJSON file with CityJSON Loader (Figure 8) in order to verify that the parsing of all city object types works. In addition, this data set validates the fact that complex LoD3 geometries can be successfully loaded in QGIS. Finally, with this file we ensured that objects with GeometryInstance representations could be loaded correctly.

## 6 DISCUSSION

In implementing this software we have faced certain challenges, the most important being the mapping of CityJSON’s hierarchical data model to QGIS’s relational data model. We have overcome this issue by providing a number of variable ways of loading city objects from a CityJSON model to QGIS. However, every difference in mapping between the two data models comes with its own compromises; in some cases information might be lost (e.g., if semantic surfaces exist in a city model but are not loaded) or data might be duplicated, resulting in redundant information due to the repetition of city‐object level attributes for multiple LoDs or semantic surfaces (when loaded as individual features).

Our approach to solving the object‐relational impedance mismatch was focused on how to import the geometries of objects so that it is optimized for the functionality provided by QGIS. This means that we intended to make the resulting layers have the minimum set of features that would be necessary in order for a user to be able to identify individual geometries, while maintaining as much semantic information as possible. Nevertheless, that choice came at the expense of hierarchical relationships between city objects. We deliberately chose not to tackle the problem of representing parent–child relationships between objects (e.g., a Building that has many BuildingParts as children) in order to avoid adding further redundancy to the resulting layers.

In our view, CityJSON parsing was proven to be straightforward and consistent when tested against several open data sets. However, a certain focus had to be put on the manipulation of GeometryInstance objects, for which inevitably some more complicated transformations have to be performed.

CityJSON Loader provides support for all CityJSON geometric types, except for MultiPoint and MultiLineString. In our experience, there are few or no CityJSON or CityGML data sets that use those geometric types. Therefore, in order to remove complexity of geometry parsing, handling, and styling we assume that all geometries in QGIS will be multi‐polygons. Nevertheless, we intend to add support for points and linestrings in the future.

From our experimentation we can conclude that QGIS 3D functionality is rather useful, especially for investigating a data set. We have tested several versions of QGIS, from 3.0 onwards, and the functionality is still improving. As of the release of version 3.10, it is possible for the user to use the 3D view not only to navigate around the geometries, but also to query specific objects by clicking on them. Furthermore, since version 3.4, conditional formatting has been available for 3D styles, which is a rather important functionality for analyzing and visualizing data by city object.

Nevertheless, the software does not provide support for the appearance module of CityJSON (i.e., colors and textures by surface). This is partly due to the limitations imposed by the 3D capabilities of QGIS, which only supports simple or rule‐based colored surfaces for 3D polygon layers.

Our experience of managing the distribution of the plugin through the QGIS main repositories is quite positive. As soon as an author has an OSGEO account (https://www.osgeo.org/community/getting‐started‐osgeo/osgeo_userid/) they can manage their plugin and version through the relevant web management page (https://plugins.qgis.org/). After a new version is uploaded, the plugin needs to be verified by the administrators of the repository. In our experience, this process did not take more than a few hours, assuming that a proper changelog was provided during the submission of a new version.

## 7 CONCLUSIONS

In this article we described the design and functionality of CityJSON Loader, our open‐source QGIS plugin for loading CityJSON. We developed this software and made it available through the official QGIS plugins repository. Furthermore, we tested the plugin by loading multiple CityJSON files in QGIS and inspecting the data loaded.

We focused on a way to solve the object‐relational mapping between CityGML and QGIS’s relational model in such a way that QGIS 3D functionality would become more efficient for the user. This means that we had to compromise on a simpler “flattened” representation of city objects in order to bring together their essential semantic information (e.g., object type and attributes) in one layer along with their geometry. We believe this is an unavoidable trade‐off in the process of loading 3D city model data into a traditional GIS data model, such as QGIS, which stores data in a tabular format. While there are certain nuances to our approach that could be further refined in the future, we believe that this approach provides the best possible way of representing 3D city model data in a GIS.

QGIS has been proven to be a useful tool for the inspection and visualization of data. The support for CRS in QGIS provides a powerful tool for the manipulation and visualization of data sets that utilize heterogeneous horizontal and vertical units (e.g., in EPSG 4979).^6^ Furthermore, QGIS provides a plethora of processing and saving tools, by incorporating most GRASS (https://grass.osgeo.org/) functions and the ability to transform data formats through GDAL (https://gdal.org/). The lack of support for textures, though, still poses limitations to the extent to which QGIS can offer visualization for some data sets.

By using CityJSON Loader against a number of open 3D city model data sets we identified a lack of metadata information in them. For instance, the Helsinki data set was missing its coordinate system definition, which forced us to manually identify it from the vendor’s website and specify the CRS during loading. We believe defining more information in the metadata of a CityJSON file would provide a better description to users regarding the nature and locality of the data. Therefore, we strongly suggest that CityJSON and CityGML data producers further focus on the subject of metadata storage.

While QGIS and similar software (such as ArcGIS) have been proven to be robust tools for proper spatial analysis over the last few decades, the rise in popularity of data sets with more complex data models is becoming a challenging task. This applies not only to CityGML, but also to other formats of data such as INSPIRE data sets, which were originally designed in UML and are also subject to object‐relational impedance mismatch. Unless a proper solution for GIS software to support more complex data structures (e.g., trees of hierarchy) is found, the manipulation of such data will remain a cumbersome process for GIS users and researchers.

In the future, we intend to enrich the functionality of the plugin by providing more tools for the manipulation of CityJSON files through QGIS. Our initial intention is to focus on the incorporation of certain processes of cjio as QGIS processing algorithms. Furthermore, we would like to investigate the possibility of exporting data in CityJSON from QGIS.

## ENDNOTES

^1^ Styling is only available from QGIS 3.2 onwards.

^2^ The C++ (https://qgis.org/api/3.10/) and Python (https://qgis.org/pyqgis/3.10/) APIs.

^3^ This feature is particularly useful for QGIS versions earlier than 3.6, where rule‐based 3D styling is not available.

^4^ This functionality is only available in versions later than QGIS 3.2 due to the technical limitations of the 3.0 API.

^5^ This functionality has only been available since QGIS 3.6 which added support for rule‐based 3D rendering styles.

^6^ EPSG 4979 is a 3D version of WGS 84, where horizontal coordinates are latitude and longitude in degrees, and vertical coordinates are height in meters (https://epsg.io/4979).
